# Long-Term Quality of Life (5-15 Years Post-Thyroidectomy) of Thyroid Carcinoma Patients in Two Tertiary Care Hospitals

**DOI:** 10.7759/cureus.22005

**Published:** 2022-02-08

**Authors:** Mohammed Yousef Alyousef, Mohammed Khaled Ghandour, Mohammed Al-Mohawes, Mosaad Alnwaisir, Tahera Islam, Khalid Al Qahtani

**Affiliations:** 1 Otolaryngology-Head and Neck Surgery, College of Medicine, King Saud University, Riyadh, SAU; 2 College of Medicine and Research Center, King Saud University, Riyadh, SAU; 3 Otolaryngology-Head &Neck Surgery, King Abdul Aziz University Hospital, King Saud University, Riyadh, SAU

**Keywords:** cancer management, thyroid carcinoma, survivorship, quality of life, thyroid cancer

## Abstract

Purpose

Early detection of thyroid cancer has reduced mortality and improved survival of patients. Increased detection has raised the incidence of early stage disease. Some physicians underestimate the suffering of these patients due to the concept of "good cancer.” The unmet needs of the survivors still need to be addressed. The objective of this paper was to evaluate the long-term quality of life (QOL) of thyroid cancer survivors.

Methods

A cross-sectional telephone survey of 211 thyroid cancer survivors who underwent thyroidectomy performed between 2006 and 2016 in two academic tertiary care hospitals was conducted using a validated Arabic version of the European Organization for Research and Treatment of Cancer head and neck cancer specific quality of life questionnaire (EROTC QLQ) - H&N43 questionnaire using a scale of 1-4, with 4 being most severe.

Results

On almost half (48.5%) of patients, thyroidectomy was performed in 2013 or earlier. Patients’ perceptions of problems were very low, with a mean score of 1.56 ± 0.7). Worry about the test results (2.37 ± 1.19), future health (2.36 ± 1.22), tingling or numbness in hands or feet (2.32 ± 1.22), pain in the shoulder (2.04 ± 1.18), and dry, itchy skin (2.04 ± 1.18) were the only items that received a mean score of more than 2. No statistically significant differences in patients’ problems were observed according to either tumor histopathology or type of thyroidectomy.

Conclusion

The overall QOL score for the patients was very good. The persistent problems identified need to be addressed in the long-term follow-up. Survivorship care plans need to be developed incorporating proper interventions.

## Introduction

Thyroid cancer (TC) is the most commonly diagnosed malignancy of the endocrine system and the ninth leading cancer in 2020. The worldwide incidence rate is three-fold higher in females than males (10.1 vs 3.1 per 100,000). Although TC accounted for 586,000 new cases worldwide in 2020, incidence of cancer-related death was substantially low (0.5 per 100.000 in women and 0.3 per 100.000 in men), with an estimated 44,000 deaths in both genders combined [1].

The recent increase in the incidence of the disease can be attributed to the early detection of papillary cancer. Concurrently, incidence of TC with stage IV has increased as well [2]. Generally, well-differentiated TC is considered as the lowest morbid solid carcinomas, with patients showing favorable long-term survival. Based on data from the Surveillance, Epidemiology, and End Results (SEER) 18 2011-2017, the five-year relative survival rate of TC patients is 98.3%. Localized tumors account for 65.9% of TC, which has an excellent prognosis of 99.9% five-year relative survival [3]. Even in patients with initial distant metastasis, the 10-year disease-specific survival rate is 56%-84.3% [4-6]. Moreover, papillary and follicular TC, which account for 80% and 10% of the TC cases, respectively [3], and has a survival rate of approximately 95% and 80%, respectively, at 30-40 years of follow-up [7,8]. The treatment for TC is mostly by surgery: total thyroidectomy with the conservation of the recurrent laryngeal nerve and parathyroid glands. This achieves disease clearance, decreases the risk of thyroid bed recurrence and, when needed, prepares the patients for adjuvant radioactive iodine therapy. Data from different studies [7-9] show that the possibility of recurrence remains even after 40 years of diagnosis and treatment. Hence, although TC is generally considered as “good cancer,” the extended and often lifelong cancer surveillance that comes with it is as anxiety provoking as any other poor prognostic cancer.

Voice alteration, swallowing dysfunction, pain, fatigue, appearance of scar, and concern about weight gain are several thyroid-specific quality of life (QOL) issues identified by previous studies [10-14]. Moreover, some patients struggle to cope with and adapt to the lifelong thyroid hormone replacement post-thyroidectomy [15]. However, despite the increased evidence of the unmet needs of the survivors in the literature [15-20], physicians tend to underestimate the suffering of these patients [21]. On top of that, very few of these studies [12,14] have addressed the long-term QOL deficit. To bridge the gap between patients’ needs and service provided [22], an effective aftercare program targeted to improve the long-term QOL of TC survivors has to be developed. To the best of our knowledge, there are no studies that have studied long-term QOL in TC patients after thyroidectomy. Our research addressed this gap in knowledge about patients’ QOL. The objective of this paper was to evaluate the long-term QOL (5-15 years of thyroidectomy) of TC survivors and identify the factors significantly associated with negative influence.

## Materials and methods

This was a quantitative, observational, cross-sectional survey. We surveyed via telephone call all thyroidectomy patients diagnosed with TC at two tertiary care hospitals between 2006 and 2016. This study included all thyroidectomy patients irrespective of whether they received post-operative adjuvant radioactive iodine ablation therapy or radical external beam radiotherapy. Patients who were treated medically only and who were not responsive to phone calls were excluded. Following the approval of the Institutional Review Board of King Saud University (approval no: KSU-IRB 017E), eligible patients were identified from operative room records and medical records. Contact information and basic demographic data such as gender, age, socioeconomic status, the specific diagnosis, and number of years after surgery were collected from the electronic health records system, retrospectively and reconfirmed during the phone survey. Participants were contacted by telephone by the investigators from January to March 2021, from Sunday to Thursday (local weekdays). The participants were asked a set of questions addressing QOL using a validated Arabic version of the European Organization for Research and Treatment of Cancer head and neck cancer specific quality of life questionnaire (EROTC QLQ) - H&N43 questionnaire [23]. Phone survey data were entered into a Microsoft Excel sheet by the investigators and later manually entered into the SPSS database (IBM Corp., Armonk, NY) by a professional statistician. The survey instrument was pilot tested on 10 patients, who were excluded from the actual sample. Verbal consent was obtained from each participant. No personal identifiers of the respondents were collected. Participants did not receive any incentive or reward for participation. In case of no response, several attempts were made to contact the patients, and after several unsuccessful contact attempts, the entry was considered invalid.

The EROTC QLQ - H&N43 questionnaire is the Head and Neck Module of the European Organization for Research and Treatment of Cancer Quality of Life Questionnaire. The Arabic version was translated, validated [24,25], and approved by the EORTC. The questionnaire consists of 43 questions incorporating multiitem symptoms related to previous week, which assess pain, swallowing problems, teeth and mouth problems, smell and taste problems, body image, eating and speech problems, cough, social interaction, sexuality, skin problems, neurological problems, wound healing, and fear of progression. The severity of the symptoms was rated on a four-point Likert scale: 1, “not at all”; 2, “a little”; 3, “quite a bit”; and 4, “very much.” Questions were selected intentionally to reflect the latent variables they were designed to measure.

Data were analyzed by the SPSS Version 30. Descriptive statistical data were presented by mean values, standard deviations, and percentages. T-tests, chi-square test, and analysis of variance (ANOVA) were used to compare subgroups. Additionally, the relationship between different variables was assessed using Pearson’s correlation. Statistical signiﬁcance is set at 0.05.

## Results

Of the 2,400 thyroid tumor patients identified and contacted, we failed to contact 1,400 due to no answer or wrong phone number. One thousand telephone calls were answered, among which 400 refused to complete the questionnaire. From the remaining 600 completed questionnaires, only 211 patients reported a history of confirmed malignant lesion. The reduced number of participants was due to our rigorous patient selection process. As the hospital electronic system was established only six years back, all the patients were diagnosed and treated before the hospital electronic system was established. Therefore, we had three steps to final selection of the patients: first step, obtaining all the patients coded with thyroid tumor from the Information Technology Department of King Saud Hospital; second step, checking all the files from the electronic medical record system and calling the patients simultaneously; and, third step, a substantial number of files did not have clear information about the patients. Therefore, diagnosis was reconfirmed with the patients during the interview.

Characteristics of participants

To determine the characteristics of participants, frequencies and percentage were used, as shown in Table 1. The vast majority of patients were diagnosed with papillary carcinoma (90.8%). Total thyroidectomy was performed on 86.9% of the patients and hemithyroidectomy on 12.6% of the patients. Majority (36.4%) of patients aged more than 50 years, while 22.7% were less than 40 years of age. Majority of patients were females (78.2%) and married (91.3%). Length of time passed after surgery showed that around half (48.5%) of patients were in the category of 2013 and older, while 17.0% of patients underwent surgery between 2005 and 2008.

**Table 1:** Sociodemographic and disease -related characteristics of thyroid cancer survivors (n=206) FTC, follicular thyroid cancer; PTC, papillary thyroid cancer

Selected Characteristics		Frequency	Percentage	
Diagnosis	FTC	16	7.8	
	Hurthle cell carcinoma	3	1.5	
	PTC	187	90.8	
Procedure	Hemithyroidectomy	26	12.6	
	Total thyroidectomy	179	86.9	
	Subtotal thyroidectomy	1	0.5	
Age	Less than 40 years	57	27.7	
	40-50 years	74	35.9	
	More than 50 years	75	36.4	
Sex	Male	45	21.8	
	Female	161	78.2	
Marital status	Single	18	8.7	
	Married	188	91.3	
Education level	Uneducated	23	11.2	
	Elementary	23	11.2	
	Middle school	25	12.1	
	High school	29	14.1	
	Bachelors	67	32.5	
	Diploma	29	14.1	
	Higher education	10	4.9	
Which year did you do the surgery?	2005-2008	35	17.0	
	2009-2012	71	34.5	
	2013 and later	100	48.5	

Patient symptoms or problems

To determine patients’ symptoms or problems, mean and standard deviation were used, as shown in Table 2. The results showed that patients’ perceptions of symptoms or problems were quite low, with a mean score of 1.56 ± 0.47. Most of the items received a mean score of 1, and none of the items scored 3. The items that received a mean of 2 or more are mentioned bellow. Item number (38) “Have you worried about the results of examinations and tests?” was the highly perceived item and ranked first, with a mean score of 2.37 ± 1.19, followed by item number (39) “Have you worried about your health in the future?” with a mean score of 2.36 ± 1.22, and, in the third place, item number (41) “Have you had tingling or numbness in your hands or feet?” with a mean score of 2.32 ± 1.22, while item number (32) “Have you had pain in your shoulder?” comes in the fourth place, with a mean score of 2.04 ± 1.18, followed by item number (34) “Have you had skin problems (e.g. itchy, dry)?” with a mean score of 2.04 ± 1.18.

**Table 2:** Mean and median scores with standard deviations of various persistent problems in thyroid cancer patients according to EROTC QLQ - H&N43 questionnaire (n=206)

No.	Items	Median	Mean ± SD
1	Have you had pain in your mouth?	1.00	1.21 ± 0.56
2	Have you had pain in your jaw?	1.00	1.29 ± 0.68
3	Have you had pain in your throat?	1.50	1.88 ± 1.04
4	Have you had problems swallowing liquids?	1.00	1.51 ± 0.91
5	Have you had problems swallowing pureed food?	1.00	1.30 ± 0.68
6	Have you had problems swallowing solid food?	1.00	1.80 ± 1.07
7	Have you choked when swallowing?	1.00	1.79 ± 1.05
8	Have you had problems with your teeth?	1.50	1.95 ± 1.13
9	Have you had problems because of losing some teeth?	1.00	1.62 ± 1.03
10	Have you had problems opening your mouth wide?	1.00	1.42 ± 0.82
11	Have you had a dry mouth?	1.00	1.74 ± 1.01
12	Have you had sticky saliva?	1.00	1.48 ± 0.86
13	Have you had problems with your sense of smell?	1.00	1.21 ± 0.61
14	Have you had problems with your sense of taste?	1.00	1.20 ± 0.60
15	Have you had problems with coughing?	1.00	1.46 ± 0.83
16	Have you had problems with hoarseness?	1.50	1.93 ± 1.10
17	Have you had problems with your appearance?	1.00	1.55 ± 0.98
18	Have you felt less physically attractive as a result of your disease or treatment?	1.00	1.52 ± 0.94
19	Have you felt dissatisfied with your body?	1.00	1.52 ± 0.93
20	Have you had problems eating?	1.00	1.40 ± 0.79
21	Have you had problems eating in front of your family?	1.00	1.18 ± 0.53
22	Have you had problems eating in front of other people?	1.00	1.29 ± 0.72
23	Have you had problems enjoying your meals?	1.00	1.24 ± 0.61
24	Have you had problems talking to other people?	1.00	1.27 ± 0.69
25	Have you had problems talking on the telephone?	1.00	1.31 ± 0.73
26	Have you had problems talking in a noisy environment?	1.00	1.57 ± 0.99
27	Have you had problems speaking clearly?	1.00	1.38 ± 0.79
28	Have you had problems going out in public?	1.00	1.26 ± 0.72
29	Have you felt less interest in sex?	1.00	1.51 ± 0.97
30	Have you felt less sexual enjoyment?	1.00	1.55 ± 0.99
31	Have you had problems raising your arm or moving it sideways?	1.00	1.61 ± 1.05
32	Have you had pain in your shoulder?	2.00	2.04 ± 1.18
33	Have you had swelling in your neck?	1.00	1.80 ± 1.04
34	Have you had skin problems (e.g., itchy, dry)?	2.00	2.02 ± 1.15
35	Have you had a rash?	1.00	1.27 ± 0.71
36	Has your skin changed color?	1.00	1.44 ± 0.84
37	Have you worried that your weight is too low?	1.00	1.29 ± 0.71
38	Have you worried about the results of examinations and tests?	2.00	2.37 ± 1.19
39	Have you worried about your health in the future?	2.00	2.36 ± 1.22
40	Have you had problems with wound healing?	1.00	1.41 ± 0.83
41	Have you had tingling or numbness in your hands or feet?	2.00	2.32 ± 1.22
42	Have you had problems chewing?	1.00	1.43 ± 0.83
Overall mean		1.42	1.56 ± 0.47

Differences according to histopathology

To determine if there were statistically significant differences in patients’ symptoms or problems according to histopathology, one-way ANOVA test was used, as shown in Table 3. There were no statistically significant differences in patients’ symptoms or problems according to histopathology (p = 0.105). The previous result indicates that there is convergence in the patients’ symptoms or problems according to histopathology.

**Table 3:** One-way ANOVA test for the differences in patients’ symptoms or problems according to histopathology (n=206) ANOVA, analysis of variance; FTC, follicular thyroid cancer; PTC, papillary thyroid cancer

	FTC (n=15)	Hurthle cell carcinoma (n=3)	PTC (n=187)	F-test	p-Value
	2.33 ± 0.20	1.35 ± 0.40	1.59 ± 0.47	2.074	0.105

Differences according to procedure

To determine if there were statistically significant differences in patients’ symptoms or problems according to type of thyroidectomy, one-way ANOVA test was used, as shown in Table 4. There were no statistically significant differences in patients’ symptoms or problems according to type of thyroidectomy (p = 0.593). The previous result indicates that there is convergence in the patients’ symptoms or problems according to procedure.

**Table 4:** One-way ANOVA test for the differences in patient symptoms or problems according to surgical procedure (n=206) ANOVA, analysis of variance

Hemithyroidectomy (n=26)	Thyroidectomy (n=179)	Subtotal thyroidectomy (n=1)	F-test	p-Value
1.53 ± 0.44	1.57 ± 0.48	1.12 ± 0.01	0.525	0.593

## Discussion

In our study, the overall QOL was found to be satisfactory to the patients. The only concerns shown by the patients were the fear from the results of the test and examinations and apprehension of future health issues. Similarly, Banach et al. reported anxiety/uncertainty about the future as the most common complaint (21.9%) among the TC patients [16]. Aschebrook-Kilfoy et al. investigated the psychological aspect of the patients’ QOL focusing on patients’ distress during different phases of the disease and treatment. Similar trend was observed in their results: distress of initial diagnosis (mean = 2.35; SD = 2.67), distress of ablation (mean= 2.84; SD = 2.82), distress from surgery (mean = 3.00; SD = 2.69), fear of a second cancer (mean = 3.77; SD = 3.11), and distress from withdrawal from thyroid hormone (mean = 3.78; SD = 3.99) scored lowest in the QOL questionnaire [22]. Likewise, Lubitz et al. reported 65% of the patients having high-level anxiety/depression in the post-operative period and during follow-up [26]. Additionally, a good number of patients (13%) experience shock, sadness, fear, frustration, or stress related to initial diagnosis, treatment, and prognosis [27].

Most of the surgery-related patient symptoms were tingling of the hand and feet, which was caused by hypocalcemia. Lubitz et al. presented open-ended questions to the patients about their concern at the time of the diagnosis of the disease. Most of the patients’ concerns were related to the complications from the surgery, change in lifestyle, the need for lifelong hormonal replacement, and the disease prognosis [26].

McIntyre et al. [10] had a result similar to us with the voice change in only 9% of the patients. However, this problem was only apparent in the first six months post-surgery. Both studies agreed that the patients were satisfied with their wound healing. Lastly, some patients complained of tingling in the first 24 hours after surgery, which was managed in the hospital, and other patients were diagnosed with osteoporosis and prescribed calcium medication. Up to our knowledge, pain in the shoulders was not measured in any other study. Strangely, in the current study, this was the fourth most common concern raised by the patients. Goldfarb and Casillas studied the QOL in young adult TC survivors; results showed that the young patients mostly complained of symptoms related to the psychological issue such as the scar after the surgery, anxiety, and headache. Other symptoms such as voice alteration and dryness of the mouth were less reported issues [11]. Surgery for TC leads to worse mental and physical QOL scores compared to the scores of the general population; however, there is a trend toward eventual recovery over time. Similar to our results, Hedman et al. recorded several persistent long-lasting health problems as did Husson et al. In Hedman et al.’s study, 238 patients out of total 279 participants reported at least one symptom from the list of fatigue, sleeping disorders, irritability, lower stress resistance, muscle weakness, bodily restlessness, sweating, palpitations, and flushes [14]. Husson et al. [28] observed muscular/joints pain (64%), chilly feeling (52%), fatigue (50%), leg cramp (43%), and hot flushes (40%) in long-term TC survivors.

In the current study, no significant relationships were observed between subtypes of TCs and QOL nor between different procedures including thyroidectomy or hemithyroidectomy and QOL. In Shah et al.’s study, total thyroidectomy patients did not demonstrate any difference in QOL compared to hemithyroidectomy patients [29]. Aschebrook-Kilfoy et al. mentioned that after five years of following up patients, QOL plateaus and then gradually increases over time [22]. This might explain the overall satisfactory QOL in our patients, as the minimum follow-up period in our study is five years. Therefore, our QOL is better. Malterling et al. studied differentiated thyroid cancer (DTC) survivors after 11.0 ± 12.5 years of diagnosis and found that the mental and physical quality QOL of treated DTC patients are similar to the healthy population [12]. Husson et al. [30] found in their systematic review that majority of the long-term survivors report several persistent thyroid-specific medical problem, even with a is satisfactory QOL score. Nickel et al. [27] reported similar results that patients diagnosed with DTC reported a wide range of QOL issues. However, their result demonstrated that the risk to have poorer QOL is 1.5 times more in case of total thyroidectomy, which is contradictory to our result. Most of the patients reported fear sadness or stress related to their health and the future or the prognosis of the disease. Schroeder et al. [31] concluded that TC patients were not significantly different from the normal population on the 36-Item Short Form Survey (SF-36) scales and on the mental scale. However, patients on thyroid hormone therapy scored better than the normal population on physical function and pain scales but scored worse on the general scale and mental health scale. Total thyroidectomy can reduce the risk of local recurrence; however, the risk of parathyroid injury and laryngeal nerve injury can greatly impact the patients’ QOL as their daily lives are changed in many ways, including increased psychological stress. The QOL of DTC patients was evaluated at one year of follow-up. DTC patients showed much lower scores on different domains except for physical functioning, which was measured using the SF-36 questionnaire [13].

One of the limitations of our study was that some of the patients were lost to follow-up and could not be interviewed because of wrong or changed contact information due to the time gap (10-15 years post-surgery). Also, central neck dissection, lateral neck dissections, radioactive iodine, and external bean radiotherapy could not be considered separately during data collection or analysis due to the scarcity of information in the very old files.

## Conclusions

In conclusion, the QOL of the TC patients was very good and satisfactory. Nevertheless, this study identified several persistent problems such as worry about the test results and future health, tingling or numbness in the hands or feet, pain in the shoulder, and dry and itchy skin. Although the problems are few in number, but by no means they are insignificant, especially considering the time frame of 5-15 years after thyroidectomy. The most common QOL issues can be predicted and discussed with the patients pre-operatively. Most importantly, these problems need to be addressed in the long-term follow-up, and, ultimately, proper survivorship care plans need to be developed with appropriate interventions targeting these specific problems.
